# Hypertension prevalence but not control varies across the spectrum of risk in patients with atrial fibrillation: A RE-LY atrial fibrillation registry sub-study

**DOI:** 10.1371/journal.pone.0226259

**Published:** 2020-01-15

**Authors:** Finlay A. McAlister, Rajibul Mian, Jonas Oldgren, Lars Wallentin, Michael Ezekowitz, Salim Yusuf, Stuart J. Connolly, Jeff S. Healey, for the RE-LY Atrial Fibrillation Registry Investigators

**Affiliations:** 1 Division of General Internal Medicine, Faculty of Medicine and Dentistry, University of Alberta, Edmonton, Canada; 2 Population Health Research Institute, McMaster University, Hamilton, Canada; 3 Uppsala Clinical Research Center and Department of Medical Sciences, Uppsala University, Uppsala, Sweden; 4 Lankenau Institute for Medical Research, Wynnewood, PA, United States of America; University of Perugia, ITALY

## Abstract

**Background:**

Although hypertension is the most common risk factor for atrial fibrillation (AF), whether blood pressure (BP) control varies across the spectrum of stroke risk in patients with AF or by adequacy of their thromboprophylaxis management is unclear.

**Methods:**

We examined data from the RE-LY AF registry conducted at 164 emergency departments (EDs) in 47 countries between December 2007 and October 2011.

**Results:**

Of the 15,400 patients in the registry, we analyzed the 9929 (mean age 67.5 years, 51.9% men) with a prior history of AF and complete BP data. While 6508 (66.5%) AF patients had hypertension, the prevalence varied widely depending on comorbidity profiles: from 45.4% in those without other cardiovascular risk factors to 82.5% in those with AF and diabetes. Although 93.9% of AF patients with hypertension were on at least one antihypertensive agent, fewer than half had BP levels ≤ 140/90 with no difference across risk profiles: 45.9% of those with NVAF and CHADS_2_ scores of 1 and 45.6% of those with NVAF and CHADS_2_ scores of 2 or more (46.9% and 45.3% for CHA_2_DS_2_-VASc scores of 1 versus 2 or more). BP control rates were not significantly better in those NVAF patients receiving guideline concordant thromboprophylaxis management (47.2%, aOR 1.03, 95%CI 0.89–1.20) than in those not receiving guideline-concordant antithrombotic therapy (45.3%).

**Conclusions:**

Hypertension was common in patients with AF but BP control rates were sub-optimal and varied little across the spectrum of stroke risk or by adequacy of thromboprophylaxis. This highlights the need for an increased focus on total atherosclerotic risk rather than just thromboprophylaxis management in AF patients.

## Introduction

Although multimorbidity is common, cardiovascular (CV) guidelines traditionally emphasize the treatment and attainment of “target levels” for individual risk factors such as hypertension, dyslipidemia, or dysglycemia which differ depending on their other comorbidities like diabetes or chronic kidney disease but without regard for other cardiovascular conditions such as atrial fibrillation.[1] The interplay between the presence of other CV risk factors and control rates for each condition is unclear as studies have reported conflicting results.[2–6] In a prior publication from the RE-LY AF registry cohort, we reported that 62% of patients presenting to emergency departments (EDs) with atrial fibrillation or flutter had hypertension (varying from 42% in India to 81% in Eastern Europe) and that 65% of those with hypertension had controlled blood pressure (BP) levels (varying from 56% in North America and Western Europe to 78% in India).[7] For the purposes of this study, we wanted to explore whether hypertension prevalence and BP treatment and control rates differed by CV risk profiles, CHADS_2_ scores, or thromboprophylaxis management in patients presenting to EDs with pre-existing atrial fibrillation (AF).

While some may question whether BP readings in an ED are accurate and reliable, two prospective cohort studies conducted in American EDs demonstrated a strong correlation between elevated ED BP levels and elevated BP at subsequent outpatient clinic visits regardless of the patient’s presenting ED symptoms.[8,9] The 63% to 81% concordance between BP readings in the ED and in the ambulatory setting is actually similar to the degree of correlation seen between first and subsequent measurements in population-based hypertension surveys in nonacute settings.[10] Furthermore, an analysis from Korean EDs confirmed that patients with elevated BP readings in the ED (regardless of their presenting complaints) exhibited higher risks of major adverse cardiovascular events in the subsequent 3 years (HR 4.25, 95% CI 3.83–4.71) and 10 years (HR 3.20, 95% CI 2.50–4.11).[11] As a result of studies such as these, the American College of Emergency Physicians did issue a clinical policy document endorsing the use of ED BP readings for screening individuals for hypertension during ED visits for any reason.[12]

## Materials and methods

We conducted this secondary analysis of data collected for the RE-LY AF registry conducted at 164 sites in 47 countries between December 2007 and October 2011, and described fully elsewhere.[7] In brief, the RE-LY AF registry enrolled consenting patients presenting to participating EDs with atrial fibrillation or flutter as the most responsible or secondary diagnosis. All diagnoses (including rheumatic vs. nonvalvular AF, presence of left ventricular hypertrophy or other comorbidities, etc) were assigned by the attending physician caring for the patient in the ED and collected on pre-standardized case report forms. Ethics approval was obtained from each participating institution (listed in the S1 Table), the study adhered to the principles of the Declaration of Helsinki, and all patients provided written signed consent. Although data sharing agreements prohibit us from making the dataset publicly available, the Population Health Research Institute has a formal data sharing policy and access may be granted to those who meet pre-specified criteria for confidential access, available by contacting information@phri.ca. Data will be disclosed only upon request and approval of the proposed use of the data by a review committee created by leaders of the study. This review will serve to ensure that patient privacy and rights, and data and research integrity, can be maintained. Review criteria will include demonstrated competence in data security and analysis and data will be shared to achieve the objectives in the approved protocol only. Individual participant data and a data dictionary will be made available, subject to requirements or restrictions from research ethics board or institutional review boards, existing contracts or agreements, and conditions set forth in participant consent forms. Data provided will be limited to data which underlies the results in the main publication after de-identification. The protocol and statistical analysis plan for analysis of the primary results will be shared. Data can be disclosed from 2 years after the main paper is published, plus 6 additional months for every year of study conduct (so from January 2018 to January 2021 for the RE-LY AF Registry). Data will be made available through secure data transfer methods overseen by the Population Health Research Institute (PHRI), or by having analyses performed by the PHRI Department of Statistics, subject to capacity. Each proposal must identify and provide funding to defray the costs of data preparation, storage, transfer, and analysis for the organization incurring these costs.

For this sub-study, as we were interested in long-term care patterns in patients with known AF, we excluded patients with a new diagnosis of AF at the time of their ED presentation but included patients even if they didn’t have a history of hypertension or elevated blood pressure in the ED. We did this as we wanted to explore whether the care of patients with AF and concomitant hypertension differed prior to their ED presentation–were physicians targeting less or more intensive BP target levels in AF patients with higher stroke risk? This sub-study was not pre-specified in the RE-LY AF registry protocol.

We defined hypertension as being present if a subject reported a health care provider-assigned diagnosis of hypertension, whether or not they were currently taking antihypertensive medications, or if their average ED blood pressure exceeded 140/90 mm Hg. We defined study participants with hypertension as having “controlled” blood pressure if the measurement in the ED revealed their SBP was < 140 mmHg and their DBP was < 90 mmHg. ED BP readings were done using a mix of automated or manual devices, depending on the centre (which method was used was not recorded).

For all patients without rheumatic heart disease (ie. those patients with NVAF), we assigned a CHADS_2_ score (and in a sensitivity analysis their CHA_2_DS_2_-VASc score) based on the comorbidities assigned by their clinician at the ED visit. Note that neither the CHADS_2_ score nor the CHA_2_DS_2_-VASc score have been validated or recommended for use in patients with rheumatic/valvular AF.

We categorized thromboprophylaxis management into 3 groups:

1. “guideline discordant” if patients with rheumatic heart disease or with nonvavular AF and CHADS_2_ scores of 1 or more were not taking warfarin or a direct oral anticoagulant (DOAC) at the time of discharge from the ED, or if patients with NVAF and CHADS_2_ scores of 0 were taking warfarin or DOAC.
2. “guideline concordant, but outside target range” if patients with rheumatic heart disease or with nonvavular AF and CHADS_2_ scores of 1 or more were taking warfarin at the time of discharge from the ED but the average of their last 3 INRs measured before and during the index ED visit was <2 or >3
3. “guideline concordant, in range” if (a) patients with rheumatic heart disease or with nonvavular AF and CHADS_2_ scores of 1 or more were on a DOAC or taking warfarin with average INR between 2 and 3 based on the last 3 INRs measured before and during the index ED visit or if (b) patients with nonvalvular AF and CHADS_2_ scores of 0 were not taking warfarin or a DOAC. The average INR approximates the Rosendaal Time-in-Therapeutic Range[13] when there are a small number of INR measurements. A TTR of at least 65% is often used as the cutpoint for defining “good INR control” since patients randomized to warfarin in the clinical trials proving the efficacy of anticoagulation had their INRs within target range 65% of the time,[14] and a post-hoc analysis of the ACTIVE-W Trial demonstrated that warfarin-treated patients with ≤65% of their INRs between 2 and 3 had higher rates of embolic and bleeding events than antiplatelet-treated patients.[15]

We compared BP control rates across pre-defined strata using chi-squared test or ANOVA as appropriate. We adjusted for age, sex, presence/absence of diabetes or LVH, and ED site/geographic region in mixed effect logistic regression analyses. P values less than 0.05 were defined as statistically significant.

## Results

The RE-LY AF registry included 15,400 patients (median age 68 years, 53% male, and 62% with a history of hypertension), but after excluding the 5451 patients with new onset AF detected for the first time at the index ED visit and the 20 without complete blood pressure data, we analyzed 9929 patients (S1 Fig).

**Fig 1:**
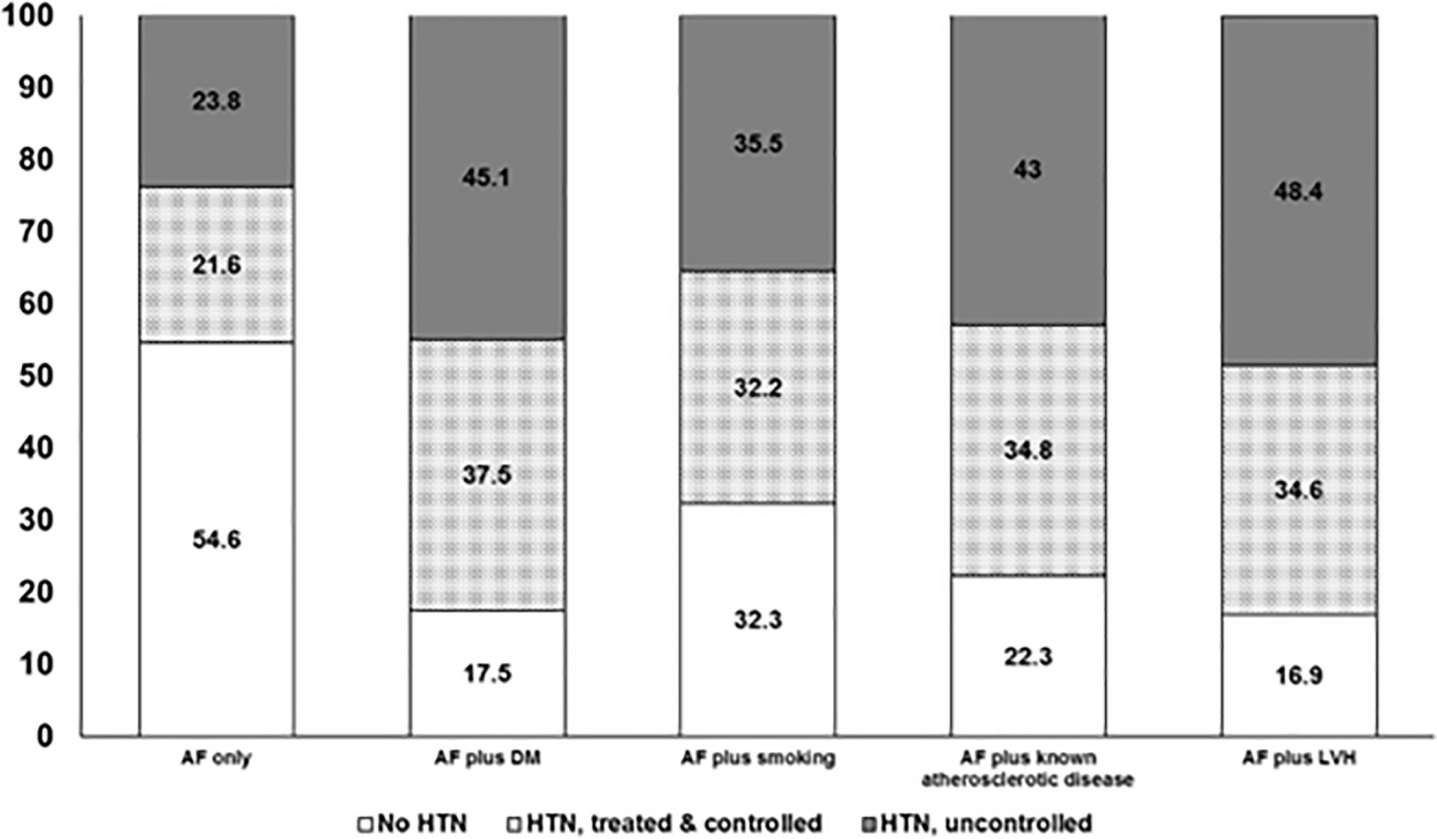
Hypertension prevalence, treatment, and control in RE-LY participants, stratified by other risk factors.

Of the 9929 patients (mean age 67.5 years, 51.9% men) presenting to participating EDs with a prior diagnosis of atrial fibrillation/flutter, 1264 (12.7%) had rheumatic heart disease, 8664 (87.3%) had NVAF (one patient was missing data on whether they had valvular disease or not), 9282 (93.5%) had atrial fibrillation confirmed on ECG, 6508 (66.5%) had hypertension, and the most common presenting complaints as assigned by the ED attending physicians were “arrhythmia, palpitations, dizziness, or syncope”, “heart failure or dyspnea”, and “chest pain” (Table 1). Patients with hypertension were significantly older and more likely to have other atherosclerotic risk factors (Table 1) than those without hypertension. The prevalence of hypertension varied widely depending on comorbidity profiles: from 45.4% of patients with AF but no other cardiovascular risk factors to 82.5% of those with AF and diabetes (p<0.0001, Fig 1). Although 93.9% (6112) of AF patients with hypertension were on at least one antihypertensive agent, less than half had controlled BP levels in the ED: 41.7% of those with LVH, 47.6% of those who smoked, 45.4% of those with diabetes, 44.7% of those with known atherosclerotic disease, and 47.6% of those without other cardiovascular risk factors beyond their AF. Mean BP levels were 122.2/75.8 mm Hg in those without hypertension, 118.1/71.3 mm Hg in those with treated and controlled hypertension, and 154.6/90.6 mm Hg in those with uncontrolled hypertension (Table 1).

**Table 1:** Patient characteristics by hypertension status.

	Overall n = 9929	Non-Hypertensive n = 3421	Hypertensive, but BP controlled n = 2984	Hypertensive, BP uncontrolled n = 3524	P value
Mean age	67.5 (14.0)	60.8 (16.5)	71.0 (11.0)	71.0 (10.9)	<0.0001
Men	5151 (51.9)	1842 (53.8)	1619 (54.3)	1690 (48.0)	<0.0001
Mean SBP	132.5 (24.9)	122.2 (20.9)	118.1 (13.9)	154.6 (19.8)	<0.0001
Mean DBP	79.7 (15.4)	75.8 (13.6)	71.3 (10.7)	90.6 (14.0)	<0.0001
On antihypertensive agent(s)	6112 (61.6)	0	2823 (94.6)	3289 (93.3)	<0.0001
On warfarin	4272 (43.0)	1409 (41.2)	1348 (45.2)	1515 (43.0)	0.006
Other Anticoagulants	1898 (19.1)	707 (20.7)	560 (18.8)	631 (17.9)	0.01
**Most Common Presenting Complaints to the ED:**					
1. Arrhythmia, palpitations, dizziness, or syncope	4620 (46.5)	1649 (48.2)	1376 (46.1)	1595 (45.3)	0.04
2. heart failure	1143 (11.5)	442 (12.9)	290 (9.7)	411 (11.7)	0.0003
3. chest pain, including acute coronary syndrome	938 (9.4)	234 (6.8)	304 (10.2)	400 (11.4)	<0.0001
4. dyspnea	908 (9.1)	378 (11.0)	263 (8.8)	267 (7.6)	<0.0001
5. infection or sepsis	374 (3.8)	128 (3.7)	142 (4.8)	104 (3.0)	0.0007
6. neurologic deficit	270 (2.7)	75 (2.2)	52 (1.7)	143 (4.1)	<0.0001
**Other Risk Factors:**					
Tobacco User	1662 (16.7)	537 (15.7)	535 (17.9)	590 (16.7)	0.06
Diabetes Mellitus	2280 (23.0)	398 (11.5)	854 (28.6)	1028 (29.2)	<0.0001
LVH by ECG or echo	2891 (29.1)	490 (14.3)	1001 (33.5)	1400 (39.7)	<0.0001
Known CAD or prior MI	3699 (37.3)	757 (22.1)	132 (4.4)	1613 (45.8)	<0.0001
Prior stroke or TIA	1592 (16.0)	366 (10.7)	550 (18.4)	676 (19.2)	<0.0001
Known atherosclerotic disease	4589 (46.2)	1022 (29.9)	1596 (53.5)	1971 (55.9)	<0.0001
Known Heart Failure	4007 (40.4)	1255 (36.7)	1251 (41.9)	1501 (42.6)	<0.0001
Dementia	429 (4.3)	77 (2.3)	191 (6.4)	161 (4.6)	<0.0001
COPD	1344 (13.5)	340 (9.9)	468 (15.7)	536 (15.2)	<0.0001
***Rheumatic heart disease (Valvular AF)***	1264 (12.7)	921 (26.9)	171 (5.7)	172 (4.9)	<0.0001
***NVAF***	8664 (87.3)	2500 (73.1)	2812 (94.2)	3352 (95.1)	<0.0001
**Stroke risk and Management in the 8664 patients with NVAF, grouped by CHADS**_**2**_ **score**					
**CHADS**_**2**_ **0**	1025 (11.8)	1025 (30.0)	0	0	<0.0001
and not on warfarin or DOAC	577 (6.7)	577 (16.9)	0	0	<0.0001
and on warfarin or DOAC	448 (5.2)	448 (13.1)	0	0	<0.0001
**CHADS**_**2**_ **1**	2202 (25.4)	871 (25.5)	611 (20.5)	720 (20.4)	<0.0001
**CHADS**_**2**_ **2 or more**	5437 (62.8)	604 (17.7)	2201 (73.8)	2632 (74.7)	<0.0001
**For All 8664 NVAF Patients and using CHADS**_**2**_ **score:**					
Thromboprophylaxis Management guideline concordant and in target range (if on warfarin, avg INR 2–3, or on any other anticoagulant)	1826 (21.1)	815 (32.6)	477 (17.0)	534 (15.9)	<0.0001
Thromboprophylaxis Management guideline concordant but outside target range (avg INR <2 or >3 if on warfarin)	3129 (36.1)	611 (24.4)	1183 (42.1)	1335 (39.8)	<0.0001
Guideline disconcordant thromboprophylaxis Management*	3709 (42.8)	1074 (43.0)	1152 (41.0)	1483 (44.2)	0.04
**Stroke risk and Management in the 8664 patients with NVAF, grouped by CHA**_**2**_**DS**_**2**_**-VASc score**					
**CHA**_**2**_**DS**_**2**_**-VASc 0**	470 (5.4)	470 (13.7)	0	0	<0.0001
and not on warfarin or DOAC	286 (3.3)	286 (8.4)	0	0	<0.0001
and on warfarin or DOAC	184 (2.1)	184 (5.4)	0	0	<0.0001
**CHA**_**2**_**DS**_**2**_**-VASc 1**	922 (10.6)	562 (16.4)	169 (5.7)	191 (5.4)	<0.0001
**CHA**_**2**_**DS**_**2**_**-VASc 2 or more**	7272 (83.9)	1468 (42.9)	2643 (88.6)	3161 (89.7)	<0.0001
**For All 8664 NVAF Patients and using CHA**_**2**_**DS**_**2**_**-VASc score:**					
Thromboprophylaxis Management guideline concordant and in target range (if on warfarin, average INR 2–3, or on any other anticoagulant)	1605 (18.5)	594 (23.8)	477 (17.0)	534 (15.9)	<0.0001
Thromboprophylaxis Management guideline concordant but outside target range (average INR <2 or >3 if on warfarin)	3323 (38.4)	805 (32.2)	1183 (42.1)	1335 (40.0)	<0.0001
Guideline disconcordant thromboprophylaxis Management**	3736 (43.1)	1101 (44.0)	1152 (41.0)	1483 (44.2)	0.02

Percentages are for columns and are in brackets. The totals for the NVAF patients grouped by CHADS_**2**_ and CHA_**2**_DS_**2**_-VASc scores excludes the 1264 patients with valvular AF and 1 patient with missing data on whether they had valvular disease or had NVAF

*NVAF CHADS_**2**_ 0 patient on anticoagulation (warfarin or DOAC) or NVAF CHADS_**2**_ ≥1 patient not on warfarin or other anticoagulants.

**NVAF CHA_**2**_DS_**2**_-VASc 0 patient on anticoagulation (warfarin or DOAC) or NVAF CHA_**2**_DS_**2**_-VASc ≥2 patient not on warfarin or other anticoagulants.

**Fig 2:**
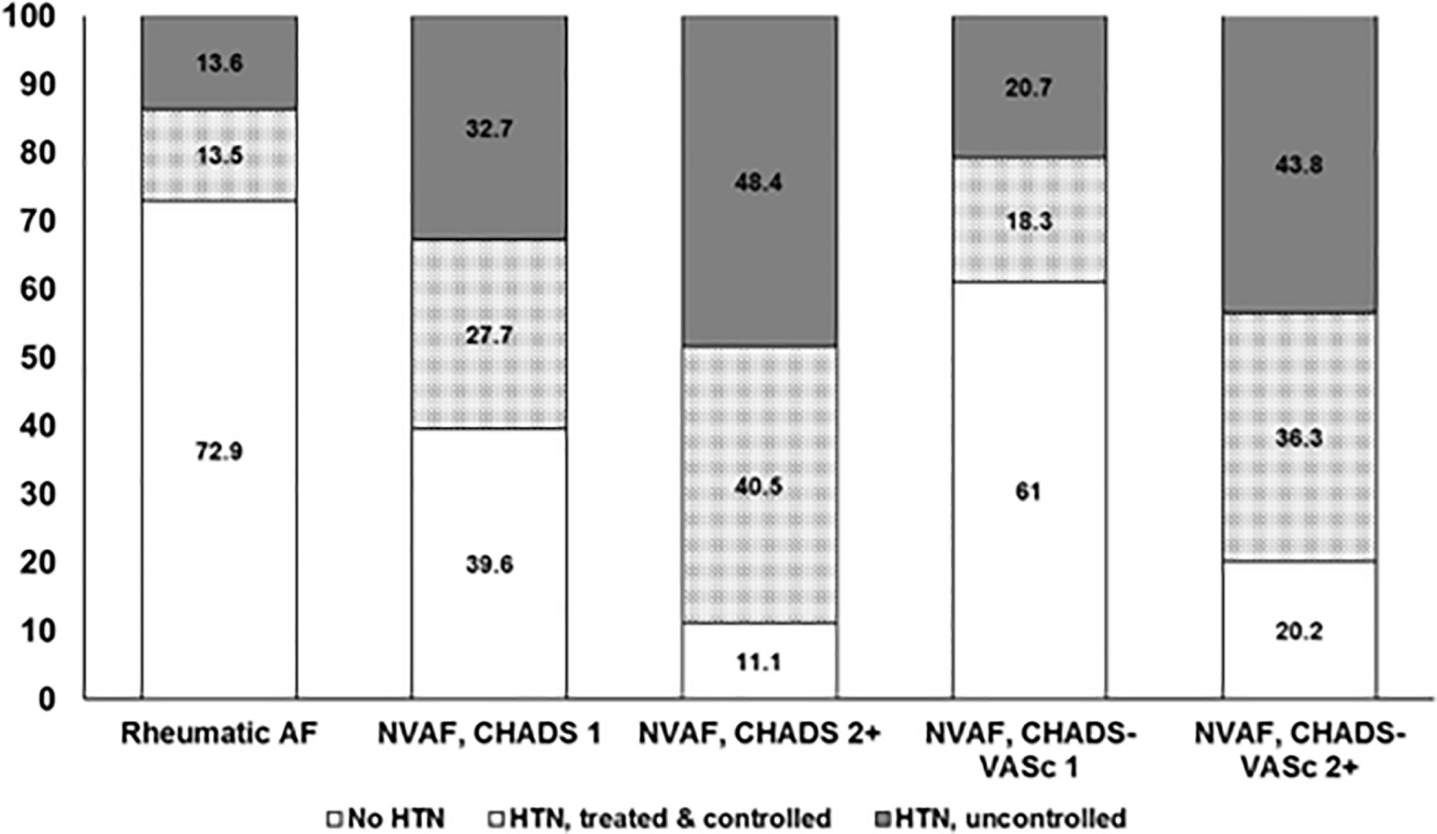
Hypertension prevalence, treatment, and control in RE-LY participants, stratified by CHADS_2_ and CHA_2_DS_2_-VASc scores.

Patients with rheumatic heart disease had a lower prevalence of hypertension (27.1%) than those with non-valvular AF (71.1%). As expected (since hypertension is one of the variables included in the CHADS_2_ score), the prevalence of hypertension varied from 0% in those NVAF patients with a CHADS_2_ score of 0 (and thus this group was left out of Fig 2) to 60.4% of those NVAF patients with a CHADS_2_ score of 1 and 89.9% of those NVAF patients with a CHADS_2_ score of 2 or more (Fig 2). Less than half of hypertensive NVAF patients had controlled blood pressure levels with little difference between those with NVAF and CHADS_2_ scores of 1 (45.9%), or those with NVAF and CHADS_2_ scores of 2 or more (45.6%, p = 0.81). Similar patterns were seen when the NVAF patients were subdivided by CHA_2_DS_2_-VASc score: 46.9% and 45.3% respectively (Fig 2).

**Fig 3:**
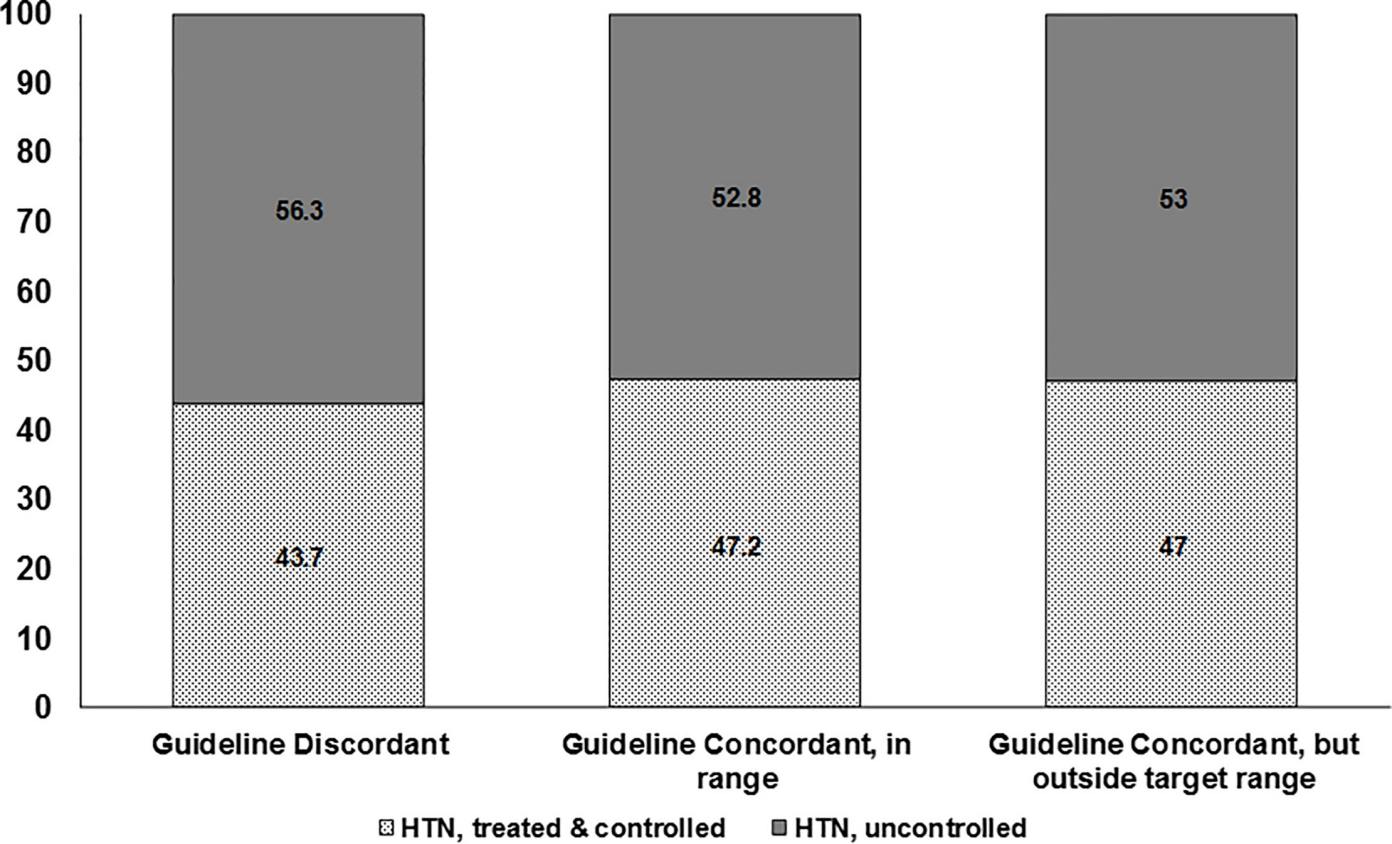
Hypertension treatment and control in RE-LY participants with NVAF, stratified by thromboprophylaxis management.

Blood pressure control rates were not significantly different between those NVAF patients with guideline concordant thromboprophylaxis management (47.2%) than in those with guideline discordant antithrombotic care (43.7%, p = 0.06, Fig 3) or those with guideline concordant antithrombotic therapy but with anticoagulation parameters outside of target range (47.0%, p = 0.91). Even after adjusting for age, sex, presence/absence of diabetes or LVH, and ED site/geographic region, there was no significant difference in BP control rates between NVAF patients receiving and not receiving guideline concordant thromboprophylaxis management (47.2% vs. 45.3%, aOR 1.03, 95%CI 0.89–1.20). Results were similar whether patients were subdivided by CHADS_2_ score or by CHA_2_DS_2_-VASc score (Table 1).

## Discussion

Our exploration of hypertension prevalence, treatment, and control rates in a cohort of patients with AF presenting to 164 EDs in all inhabited continents had three main findings.

First, hypertension was more than twice as common in patients with NVAF as in those with rheumatic disease, and hypertension prevalence increased as the number of other CV risk factors and age increased. This mirrors findings in other population-based surveys[16,17] and reflects the different pathophysiologies of valvular and NVAF.

Second, less than half of AF patients with hypertension have their BP treated and controlled to currently recommended targets, providing one potential explanation for why anticoagulated AF patients still suffer higher than expected rates of ischemic strokes and TIAs than age- and sex-matched peers without AF.[18]. The fact that there was no difference in BP control rates across NVAF strata defined by CHADS scores argues against an overt risk-treatment paradox,[2] although it could certainly be argued that patients with multiple risk factors require even more stringent attention to BP control given greater potential absolute benefits than those at lower risk for CV outcomes.

Third, hypertensive NVAF patients with guideline concordant thromboprophylaxis management were only marginally more likely to have their BP controlled than those whose thromboprophylaxis management was not guideline concordant and this was not statistically significant after multivariable analysis. This is consistent with previous studies reporting that BP control rates were not better in patients with higher atherosclerotic risk profiles[2,4,6] and other studies demonstrating that treated hypertensives have poorer prognoses than untreated normotensives with the same blood pressures due to under-treatment of their other atherosclerotic risk factors.[19–22] This is another example that concordance with one set of clinical guidelines does not predict concordance with other pertinent CV risk reduction guidelines.

Although our study is drawn from a large cohort of unselected AF patients presenting to 164 EDs and employed standardized case definitions, there are some limitations to our analysis. Most importantly, the BP measurements were done by clinicians in ED settings and did not include multiple measurements over time as currently recommended in hypertension guidelines; thus we cannot adjust for white coat effect nor detect masked hypertension. However, as pointed out in the Introduction to this paper, prospective cohort studies have established that ED BP readings are both correlated with subsequent values in ambulatory clinics and are associated with subsequent cardiovascular prognosis–thus, the American College of Emergency Physicians does endorse the use of ED BP readings for screening and decisions about management for hypertension.[8–12] It is worth noting that many population-based surveys (such as the Canadian Health Measures Survey and the National Health And Nutrition Examination Survey) also only use one reading done on one day to classify participant BP status.[3] However, we readily acknowledge that BP readings vary over time, with standard deviations as high as 12/8 mm Hg between days, that BP often falls with repeated measurement due to habituation and regression to the mean, and that ambulatory BP readings provide a more accurate picture of an individual’s true BP.[23] Thus, we believe that readers should focus on the relative comparison between AF subgroups in our study rather than absolute BP readings when interpreting our data; concerns with the use of ED BP readings is mitigated since all AF subgroups had their BPs measured in the same fashion and in the same environment and our interest was in comparing BPs between AF subgroups rather than defining population-based BP values.

A second limitation is that we assumed the target INR ranges for warfarin-treated patients were 2–3, but recognize that for a small proportion of AF patients a higher (or lower) range may be targeted clinically based on local guidelines or if patients have had thromboembolic (or bleeding) events when INR was between 2 and 3. Third, we used average INRs to define control rather than TTR. While the average INR does approximate the TTR when there are only 3 measurements (RE-LY AF only collected 3 for each participant)[13], we do acknowledge that use of the average INR may be less accurate if at least one of the INRs is substantially different from the others. Fourth, although we defined NVAF patients with CHADS scores of 0 receiving anticoagulation as being “guideline discordant”, this may not have always been the case as some of this group might include patients with subacute onset of AF scheduled for cardioversion in the near future, and thus properly pre-treated with oral anticoagulation consistent with guidelines. Fifth, as we did not have data on serum creatinines or body weight we were not able to examine whether patients taking DOAC agents were on appropriate doses. As DOAC use has increased since this study was done, concordance with guideline recommendations may be different in future years. Sixth, we used a BP level of 140/90 mm Hg to define control; use of more stringent criteria like 130/80 mm Hg would result in even lower control rates than we report. Seventh, we did not adjust for setting or clinician in our analyses but this was not possible given the large number of EDs with relatively modest numbers of AF patients from each ED. However, we did adjust for geographic region in multivariate analyses since large global variations in etiology and management have previously been demonstrated in the RE-LY registry and other international cohort studies.[24,25] Finally, as we only had access to the RE-LY registry baseline data for this analysis we were unable to examine long-term outcomes in these patients.

## Conclusion

In conclusion, while 2/3 of people with AF have hypertension and nearly 94% were taking antihypertensive medications, control rates were sub-optimal and in patients with NVAF did not vary appreciably across CHADS_2_ scores or by adequacy of thromboprophylaxis. Although most of the educational messaging for physicians and patients about AF focuses on thromboprophylaxis management, our data suggests the need for increased attention to other atherosclerotic risk factors such as hypertension, particularly since non-stroke cardiovascular outcomes are more common than stroke in AF patients.[26,27]

## Supporting information

S1 Fig Derivation of study sample from the RE-LY cohort. (TIFF)

S1 Table RE-LY AF Registry—Site/Investigator List. (DOCX)
