# Supplementary figures and images for: Hypertension prevalence but not control varies across the spectrum of risk in patients with atrial fibrillation: A RE-LY atrial fibrillation registry sub-study
Source: PLoS One. 2020 Jan 15;15(1):e0226259. doi: 10.1371/journal.pone.0226259 (PMC6961825; doi:10.1371/journal.pone.0226259)

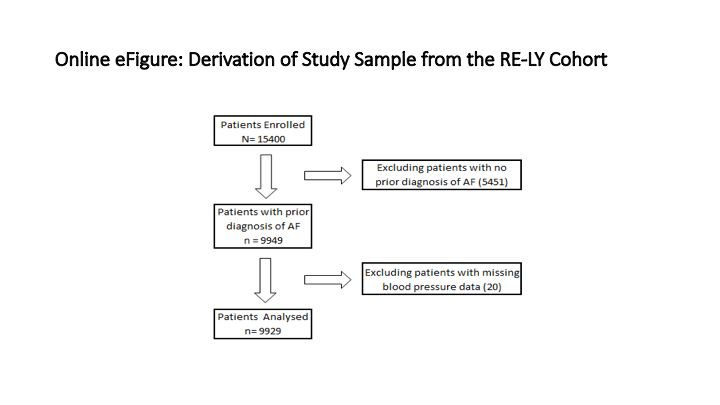

Supplement: S1 Fig — (TIFF) [file pone.0226259.s001.tiff]
