# Supplementary material for: Hypertension prevalence but not control varies across the spectrum of risk in patients with atrial fibrillation: A RE-LY atrial fibrillation registry sub-study
Source: PLoS One. 2020 Jan 15;15(1):e0226259. doi: 10.1371/journal.pone.0226259 (PMC6961825; doi:10.1371/journal.pone.0226259)
Supplement: S1 Table — (DOCX) [file pone.0226259.s002.docx]

| RE-LY AF Registry - Site/Investigator List | | |
| --- | --- | --- |
|  |  |  |
| **Country** | **Lead Investigator** | **Centre Name** |
| US | Dr. Eve Gillespie | Glacier View Cardiology PC |
| US | Dr. Ranchhod Khant | Bay Area Cardiology |
| US | Dr. Ajit Rasinghani | UCSD Medical Centre |
| US | Dr. Vuong Duthinh | Genesys Regional Medical Center |
| US | Dr. Ihsan Haque | City Cardiology |
| US | Dr. Craig McPherson | Bridgeport Hospital |
| US | Dr. Allan Murphy | Cardiovascular Center of Hampton Roads Inc |
| US | Dr. Alan Storrow | Department of Emergency Medicine |
| US | Dr. Wayne N. Leimbach Jr. | Oklahoma Heart Institute |
| US | Dr. Joel Rubenstein / Dr. Huckins (in ER) | Newton Wellesley Cardiology |
| US | Dr. Marcus Williams | The Valley Hospital |
| US | Dr. Ranchhod Khant | South Bay Hospital |
| Australia | Dr Bhuwan Singh | Launceston General Hospital |
| Argentina | Dr. Alejandro Sanchez | Policlinico Modelo Cipolletti |
| Argentina | Dr. Alberto Caccavo | Clinica Coronel Suarez |
| Argentina | Dr. Facundo Risso | Instituto de Cardilogia SRL |
| Australia | Dr. Fritz Freihoff | Kaiser-Franz-Josef-Spital Wien |
| Bulgaria | Assoc. Prof. Dimitar Raev | Medical Institue at the Ministry of Interior |
| Bulgaria | Assoc. Prof. Nina Gocheva | National Heart Hospital |
| Brazil | Dr. Jose Francisco Kerr Saraiv | Hospital e Maternidade Celso Pierro |
| Brazil | Dra. Lilia Nigro Maia | Fundacao Faculdade Regional de Medicina |
| Brazil | Dr. Adalberto Lorga Filho | Instituto de Molestia Caridovasculares |
| Brazil | Dr. Dalton Precoma | Irmandade da Santa Casa de Misericordia de Curitiba - PUC |
| Brazil | Dr. Fernando Tallo | Guarulhos Sao Paulo |
| Brazil | Dr. Ritt Luiz Eduardo | Instituto Cardiopulmonar |
| Brazil | Dr. Fabio Rossi dos Santos | Clinica Bastos |
| Brazil | Dr. Wladmir Faustino Saporito | Hospital Estadual Mario Covas |
| Brazil | Dr. Carlos Antonio A. Kalil | Hospital Sao Lucas da PUCRS |
| Brazil | Dr. Paulo Ernesto | Irmandade da Santa Casa de Misericordia |
| Brazil | Dr. Helder Reis | Fundacao Hospital de Clinicas Gaspar Viana |
| Brazil | Dr. Antonio Carlos Sobral Souza | Hospital Sao Lucas Medico Hospitalar LTDA |
| Brazil | Dr. Luciana Armaganijan | Instituto Dante Pazzanese de Cardiologia |
| Canada | Dr. Paul Dorian | St. Michaels Hospital Arrythmia Services |
| Canada | Dr. Christopher Lai | Curans Health Centre Corp. |
| Canada | Dr. Carlos Morillo | Hamilton Health Sciences |
| Canada | Dr. Gilles O'Hara | Hospital Laval Cardiology Department |
| Canada | Dr. Ratika Parkash | QEII Health Sciences Centre Cardiology |
| Canada | Dr. Kevin Pistawka Dr. Michelle Mantle | Kelowna Cardiology Research |
| Canada | Dr. David Cleveland | Penticton General Hospital |
| China | Dr. Yang Yanmin | Fu Wai Hospital |
| China | Dr.Chunsheng Li | Beijing Chao Yang Hopital |
| China | Dr.Xioajuan Bai | The First Hospital Affliated to China Me |
| China | Dr. Fang Zhang | Qingdao Municipal Hospital |
| China | Dr.Junxia Li | Bethune International Peace Hospital |
| China | Dr. Fengru Zhang | Ruijin Hospital School of Medicine Shanghai Jiaotong University |
| China | Dr.Han Sheng Tang | Wuhan Center hospital |
| China | Dr.Ji Yuan Han | Wuhan Xie He Hospital |
| China | Dr.Qing He | West China Hospital |
| China | Dr.Hongke Zeng | Guangdong People's Hospital |
| China | Dr.Jianhua Lu | Guan Zhou First Hospital |
| China | Dr.Shuging Wang | Qigzhaer First Hospital |
| China | Dr. Li Zhao | Fluxing Hospital Affliated Capital Munic |
| China | Dr.Hua Zhang | East District Branch of Quingdao Muncipa |
| China | Dr.Pengfei Yu | Pingdu Municipal Hospital |
| Colombia | Dr. Franklin Roberto Quiros Di | Fundacion Cardiovascular De Colombia |
| Colombia | Dr. Luis Fernando Pava | Fundacion Valle del Lili |
| Czech Republic | Dr. Durdil Vaclav | Faculty Hospital: Motol |
| Czech Republic | Dr. Jan Kral | Hospital Nachod |
| Czech Republic | Dr. Jiri Bednar | Hospital Trebic |
| Czech Republic | Dr. Antonin Mandovec | Nemocnice Horovice |
| Czech Republic | Dr. Oldrich Hejhal | Kromerizska Nemocnice A.S. |
| Denmark | Dr. Axel Brandes | Odense University Hospital |
| Ireland | Dr. Ricky Sheahan | Beaumont Hospital |
| Germany | Dr. Thomas Horacek | Evangelisches Krankenhaus Witten |
| Germany | Prof. Dr. Job Harenberg | IV. Medizinische Klinik Haus 8 Ebene 5 Zimmer 3 |
| Germany | Dr. Karl-Friedrich Appel | Ambulantes Herzzentrum Kassel |
| Germany | Dr. Ursula Hoffmann | University Hospital Manheim |
| Hungary | Dr. Katalin Keltai | III. Dept. of Medicine Semmelweis University |
| Hungary | Dr. Erno Kis | Janos Balassa Hospital |
| Hungary | Prof. Laszlo Csiba | Debreceni Egyetem Oec Neurolgiai Klinika |
| India | Dr R.K Agarwal | Christain Medical College , Ludhiana |
| India | Dr.J P S Sawhney | Sir Ganga Ram Hospital, N.Delhi |
| India | Dr. Sudeep Kumar | Sanjay Gandhi PGIMS, Lucknow |
| India | Dr.Shrenik Shah | Sterling Hospital, Ahmedabad |
| India | Dr. Ajay Naik | Heart Care Clinic, Ahmedabad |
| India | Dr. Anil Bharani | MGM Medical College , Indore |
| India | Dr. Archana Jain | Avanti Institute of Cardiology, Nagpur |
| India | Dr.Ramesh Babu | Medwin Hospital, Hyderabad |
| India | Dr.Kiron Varghese | St.John's Medical College,Bangalore |
| India | Dr.Johny Joseph | Caritas Hospital,Kottayam |
| India | Dr. Jagdish Hiremath | Poona Hospital, Pune |
| India | Dr.RB Panwar | S.P. Medical College , Bikaner |
| India | Dr.Arun Srinivas | Vikram Hospital & Heart Care, Mysore |
| India | Dr.J B Gupta | S. R. Kalla Memorial General Hospt, Jaip |
| Italy | Dr. Alessandro Salvioni | Centro Cardiologico Monzino |
| Italy | Dr.Daneile Coen | Niguarda Hospital |
| Japan | Dr. Eiichi Watanabe | Fujita Health University School of Med |
| Netherlands | Dr. JVM van Eck | Jeroen Bosch Ziekenhuis locatie Groot Ziekengasthuis |
| Netherlands | Dr. Isabelle van Gelder | AZG-UMCG |
| Poland | Dr. Pawel Miekus | Szpital Miejski Oddzial Kardiologiczny |
| Russia | Dr. Sergey S. Yakushin | Ryazan Regional Clinical Hospital |
| Singapore | Dr. Swee Han Lim | National Heart Centre |
| Singapore | Assoc Prof Shirley Ooi Beng Suat | National Univeristy Hospital |
| South Africa | Prof. Patrick Commerford | Groote Schuur Hospital |
| South Africa | Dr. AKM Nowshad Alam | Livigstone Hospital |
| South Africa | Prof. Karen Sliwa | Chris Hani Baragwanath Hospital |
| South Korea | Professor Kim Yoon Nyun | Keimyung University Dongsan Medical Center |
| South Korea | Professor Cho Jeong-Gwan | Chonnam National University Hospital |
| South Korea | Professor Tai Ho Rho | St. Pauls Hospital |
| South Korea | Prof. June-Soo Kim | Samsung Medical Center |
| South Korea | Prof. Nam-Ho Kim | Wonkwang University School of Medicine & |
| South Korea | Dr. Man-Young Lee | St. Mary's Hospital |
| South Korea | Prof. Seong-Ho Hu | Daejeon St. Mary's Hospital |
| Spain | Dr. Gabriel Gusi Tragant | Corporacio Sanitaria Parc Tauli |
| Sweden | Dr. Johan Herlitz | Sahlgrenska Sjukhuset |
| Sweden | Dr. Ziad Hijazi | UCR/Uppsala Kliniska Forskningscentrum |
| Sweden | Dr. Lennart Malmqvist | Kopings lasarett |
| Sweden | Dr. Peter J. Svensson | Department of Coagulation Disorders |
| Thailand | Assoc. Prof Supachai Tanomsup | Madhidol University |
| Turkey | Dr. Bunyamin Yavuz | Kecioren Teaching and Research Hospital |
| Latvia | Dr. Galina Dormidontova | Daugavpils Regional Hospital |
| Latvia | Dr. Arveds Apinis | SIA Rigas Austrumu Kliniska universitate |
| Ukraine | Dr. Olena Grishyna | Mechnikov Institute of Microbiology and |
| Ukraine | Dr. Oleksiy Onyshchenko | Donetsk National Medical University |
| Latvia | Dr. Ilja Zakke | Paul Stradins Clinical University Hospit |
| United Kingdom | Dr. Andrew Moriarty | Craigavon Area Hospital |
| United Kingdom | Dr. Maurice Pye | York District Hospital |
| Nigeria | Dr. Dike Ojji | University of Abuja Teaching Hospital |
| Nigeria | Dr. Aje Akinyemi | University College Hospital Ibadan |
| Nigeria | Dr. Mahmoud U. Sani / Dr. KM Karaye | Aminu Kano Teaching Hospital |
| Nigeria | Prof. Solomon S. Danbauchi | Ahmadu Bellow Univeristy Teaching Hospital |
| Nigeria | Dr. Okechukwu Samuel Ogah | Federal Medical Centre Idi-Aba |
| Nigeria | Dr. Victor Ansa | University of Calabar Teaching Hospital |
| Nigeria | Dr. Ejim Emmanuel | University of Nigeria Teaching Hospital |
| Nigeria | Dr. Moddy Janabi | Muhimbili National Hospital |
| Senegal | Dr. Serigne Abdou | University Hospital of Aristide Le Dante |
| Egypt | Dr. Islam Shawky | Al Farouk Hospital |
| Egypt | Dr. Ahmed Fathy Tamara | Egypt Heart Cath |
| Uganda | Dr. Mondo Charles Kiiza | Uganda Heart Institute, Mulago Hospital |
| Cameroon | Dr. Marie Ntep-Gweth | Hopital Central de Yaounde |
| Cameroon | Dr. Anastase DZUDIE TAMDJA | Douala General Hospital |
| Kenya | Dr. Gerald Yonga | Aga Khan Univeristy Hospital |
| Zambia | Dr. Fastone Goma | The University Teaching Hospital |
| Mozambique | Dr. Albertino Demasceno | Servico de Cariologia |
| Mozambique | Dr. Ana Olga H Mocumbi | Instituto do Coracao |
| United Arab Emirates | Dr. Afzal Hussein Yusufali | Dubai Hospital |
| United Arab Emirates | Dr. Wael Almahmeed | Sheikh Khalifa Medical City |
| United Arab Emirates | Dr. Ghazi Ahmad Radaidah | Rashid Hospital |
| Saudi Arabia | Dr. Bander Al Ghamdi | King Faisal Specialist Hospital and Rese |
| Iran | Dr. M.R. Mohammad Hasani | Shariati Hospital |
| China | Dr. Xiaoping Hui | Pu Dong Xin Qu Gong Li Hospital |
| China | Dr. Shiping Liu | Chuan Bei Medical College Affiliated Hos |
| China | Dr. Zhang Xi Zhou | Yichang First Hospital |
| China | Dr. Qiang Zhang | Chengdu Sixth Hospital |
| China | Dr. Zhaoxia Zan | Langfan Fourth People's Hospital |
| India | Dr. R K Premchand Jain | Krishna Institute of Medical Sciences, S |
| India | Dr. Sunil Diwedi | Workhardt Hospital, Bangalore |
| India | Dr. Sudhir Verma | Sadbhavna Madical & Heart institute, Pat |
| \| India \| \| --- \| | Dr. Brian Pinto | Holy Family Hospital, Mumbai |
| India | Dr.Tejas Patel | Krishna Heart and Superspeciality Instit |
| India | Dr. Darshan Banker | Banker Heart Center, Vadodara |
| India | Dr. Praveen Chandra | Max Devki Devi Heart & Vascular Institut |
| India | Dr. Skand Kumar | Bhopal Memorial Hospital |
| Ecuador | Dr. Rene Vicuna | Hospital Metropolitano |
| Venezuela | Dr. Ivan Mendoza | Instituto De Clinicas Y Urologia Tananac |
| Chile | Dr. Fernando Lanas | Centro De Estudios Cardiolgicos Y De Medicina Interna Ltda. |
| Chile | Dr. Paolo Varleta | Hospital Dipreca |
| Chile | Dr. Claudio Marcelo | Clinical Alemana de Puerto Varas |
| Czech Republic | MUDr. Josef Jakabcin | Krajska zdravotni a.s. |
| Czech Republic | Dr. Zbunek Jelinek | Hospital Hranice |
| Czech Republic | prim.MUDr. Hana Grunfeldova | Mestska nemocnice |
| Slovakia | Dr. Jaroslava Strbova | Kardio-Sanus, s.r.o. |
| Slovakia | Dr. Daniela Vinanska | Medispol, s.r.o |
| Sudan | Dr. Ahmed Suliman | Alshaab Teaching Hospital |
